# Blood glucose may be another index to initiate insulin treatment besides glycated hemoglobin A1c after oral antidiabetic medications failure for glycemic control: A real–world survey

**DOI:** 10.3389/fendo.2022.998210

**Published:** 2022-11-24

**Authors:** Yanli Li, Yan Wu, Yi Shu, Shu Li, Jianhao Pei, Hong Chen, Shiping Liu, Guangda Xiang, Wenbo Wang, Pengfei Shan, Heng Su, Xiaoyan Wu, Dewen Yan, Wangen Li

**Affiliations:** ^1^ Department of Endocrinology, The Second Affiliated Hospital of Guangzhou Medical University, Guangzhou, China; ^2^ Department of Endocrinology, Shenzhen People’s Hospital, Shenzhen, China; ^3^ Department of Endocrinology, The Sixth Affiliated Hospital of South China University of Technology, Foshan, China; ^4^ Department of Endocrinology, Huizhou Municipal Central Hospital, Huizhou, China; ^5^ Department of Endocrinology, Guangdong Provincial People’s Hospital, Guangzhou, China; ^6^ Department of Endocrinology, Zhujiang Hospital, Southern Medical University, Guangzhou, China; ^7^ Department of Metabolism and Endocrinology, The Second Xiangya Hospital of Central South University, Changsha, China; ^8^ Department of Endocrinology, General Hospital of Central Theater Command, Wuhan, China; ^9^ Department of Endocrinology, Peking University Shougang Hospital, Beijing, China; ^10^ Department of Endocrinology and Metabolism, The Second Affiliated Hospital of Zhejiang University School of Medicine, Hangzhou, China; ^11^ Department of Endocrinology, The Affiliated Hospital of Kunming University of Science and Technology, Kunming, China; ^12^ Department of Endocrinology, The First Affiliated Hospital of Xi’an Jiaotong University, Xi’an, China; ^13^ Department of Endocrinology, The First Affiliated Hospital of Shenzhen University, Shenzhen, China

**Keywords:** initiating insulin, glycated hemoglobin A1c, blood glucose, type 2 diabetes, inertia

## Abstract

**Objective:**

The inertia of insulin initiation is a barrier to achieving glycemic control when oral antidiabetic drugs fail to control glucose during the treatment of type 2 diabetes (T2D). Insulin initiation is usually based on glycated hemoglobin A1c (A1C). To investigate whether there is another index for insulin initiation besides A1C, we conducted a cross-sectional survey in the real world.

**Methods:**

We conducted a multicenter cross-section survey with a total of 1034 T2D patients. All patients, at the time of the survey, decided to initiate insulin therapy due to failure of controlling glucose using only oral antidiabetic drugs. We analyzed the differences of blood glucose between patients who were tested for A1C and those who were not.

**Results:**

666 (64.4%) patients were tested A1C and 368 (35.6%) were not. Neither fasting blood glucose (FBG) (12.0 ± 2.9 vs 12.3 ± 2.9 mmol/L, t = 1.494, *P* = 0.135) nor postprandial blood glucose (PBG) (18.4 ± 4.8 vs 17.9 ± 4.8 mmol/L, t = 1.315, *P* = 0.189) were significantly different between patients with and without A1C.

**Conclusion:**

Our results demonstrated that initiating insulin based on FBG or PBG is a common clinical practice, at least in China; moreover, since it is easier to obtain than A1C, it can be a simple and effective way to overcome clinical inertia for initiating insulin.

## Introduction

Glycated hemoglobin A1c (A1C) is the “gold standard” for the assessment of glycemic control over the previous 2 to 3 months, especially after the publication of Diabetes Control and Complications Trial (DCCT) and UK Prospective Diabetes Study (UKPDS) (1, 2). The American Association of Clinical Endocrinologists (AACE), the American Diabetes Association (ADA), the Chinese Diabetes Society (CDS), and the National Institute for Health and Care Excellence (NICE) recommend that the target of A1C was 6.5%, 7.0%, 7.0% and 7.5%, respectively (3–6). They recommend that additional actions should be taken when A1C exceeds the glycemic goal. These actions include initiating insulin when the maintenance of glycemic targets with oral antidiabetic drugs (OAD) therapy is no longer possible. Even if new better OAD (e.g. sodium-glucose cotransporter 2 inhibitors) and injectable GLP-1RA are available, many patients eventually require and benefit from insulin therapy (4).However, initiating insulin is the most difficult step in the treatment of type 2 diabetes (T2D) because of clinical inertia, which is a common phenomenon all over the world (7, 8). Previous studies have shown that actual A1C levels at insulin initiation far exceed the aforementioned recommended target. An international observational study involving 10 countries and 17 374 participants showed that the proportion of patients with A1C ≥9.0% ranged from 23% to 64% when initiating insulin after OAD failure (9). Recently, a survey including 18 995 patients with T2D was conducted in China and showed that patients initiate insulin when A1C was over 9.6% (10). Looking for ways to overcome the clinical inertia of initiating insulin in the treatment of T2D has been a great challenge.

Physician–, patient– and healthcare–system–related factors all contribute to clinical inertia (11). These include hypoglycemia, weight gain, adherence, indications of failure, burdensome regimens, fear of injections and/or fear of self–measuring blood glucose, worsened quality of life, and lack of experience and time (12). In addition, A1C test is time-consuming, complicated and expensive. Furthermore, the measurement of A1C usually varies between different hospitals.

Previous studies have demonstrated that rapid on-site A1C measurements may play an important role in improving glycemic control and facilitating diabetes management (13). However, most hospitals do not routinely take the A1C test in clinical practice (14). ADA and European Association for the Study of Diabetes (EASD) consensus stated that patient regular self-monitoring of blood glucose (SMBG) may help with self-management and medication adjustment, particularly in individuals taking insulin (4). A standard approach for titrating optimal basal insulin dosage is based on FBG, which is a simple and effective index (15). If PBG increment is over 3 mmol/L premix insulin is preferable (16). Therefore, initiating insulin based on FBG and/or PBG instead of A1C could be a feasible approach (4). In clinical practice, many physicians initiate insulin therapy based on blood glucose when T2D patients are not achieving glycemic goals with OAD. Here, we presume that initiating insulin based on A1C itself may be a factor contributing to insulin initiation inertia. Therefore, to investigate whether there are other indices for insulin initiation besides A1C for the treatment of T2D, we conducted a multicenter cross-sectional survey in the real world.

## Participants and methods

### Participants

This multicenter cross-section survey was conducted at 13 tertiary care medical centers in five regions of China from January 2021 to June 2021. Each center enrolled 100 patients with T2D who were not achieving glycemic goals with OAD. We recorded the following data when a patient first initiated insulin in addition to OAD: the latest fingertip FBG and/or PBG and A1C during the past three months, and his/her anthropometric and metabolic characteristics. Informed consent was obtained from each participant. This study was approved by the Ethics Committee of our hospital.

### Statistical analyses

Significance of differences of all variables between patients with and without A1C was analyzed using the Pearson’s χ^2^ and Fisher’s exact tests or Student’s t-test. Results are expressed as mean ± SD. Statistical analysis was performed using SPSS Statistical Software 25.0 (IBM Corporation, Armonk, NY, USA). *P* < 0.05 was considered statistically significant.

## Results

### Clinical characteristics of participants

A total of 1 034 patients with complete clinical data were analyzed. They had an age of 55.6 ± 9.6 years, with diabetes duration of 6.9 ± 3.9 years, and a BMI 23.6 ± 2.7kg/m^2^. Of which, 666 (64.4%) patients tested A1C and 368 (35.6%) did not. The demographic and clinical characteristics of the included subjects were presented in **Table 1**.

**Table 1:** Clinical characteristics of patients.

	With A1C	Without A1C	*x* ^2^/t	*P* value
n	666	368		
A1C (%)	9.8 ± 1.6			
Male (%))	320 (48.8)	190 (51.6)	1.217	0.270
Age (Years)	54.8 ± 9.9	57.1 ± 8.7	3.714	0.000
History (Years)	6.6 ± 3.8	7.6 ± 3.8	4.128	0.000
BMI (kg/m^2^)	23.6 ± 2.7	23.6 ± 2.5	0.267	0.789
Insulin regimen				
Basal (%)	438 (65.8)	268 (72.8)	5.455	0.020
Premix (%)	228 (34.2)	100 (27.2)	5.455	0.020

BMI, body mass index.

### Patients’ FBG and PBG levels at insulin initiation

**Figure 1:**
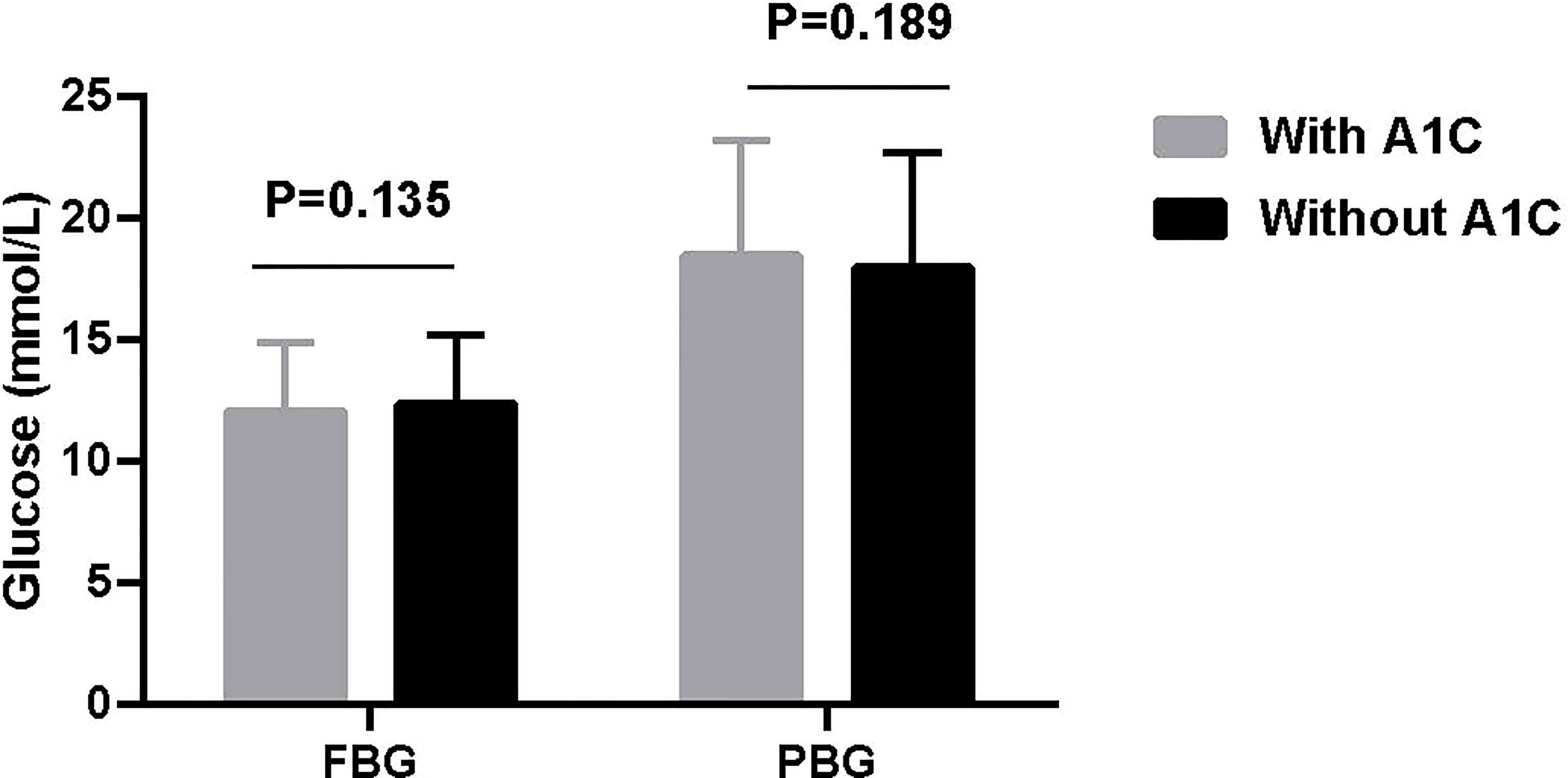
Patients’ FBG and PBG levels at insulin initiation.

In the total 1 034 patients, FBG was 12.1 ± 2.9 mmol/L and PBG was 18.2 ± 4.8 mmol/L. Further, neither FBG (12.0 ± 2.9 vs 12.3 ± 2.9 mmol/L, t = 1.494, *P* = 0.135. 95% CI, -0.0876, 0.6473) nor PBG (18.4 ± 4.8 vs 17.9 ± 4.8 mmol/L, t = 1.315, *P* = 0.189. 95%CI, -0.0867, 0.6464) were significantly different between patients with and without A1C (**Figure 1**).

## Discussion

This survey indicates that the inertia of initiating insulin still exists because our patients on average have an A1C level of 9.8%, which is much higher than A1C target of 7.0% recommended by CDS guidelines (5).

Our survey also demonstrated that more than one third of the surveyed physicians in China initiated insulin therapy for T2D treatment without testing A1C, although testing A1C is recommended by all guidelines (3–6).

Interestingly, neither FBG nor PBG were significantly different between patients with and without A1C. Besides, the P value is far from 0.05 and the sample size is big enough which further confirm the reliability. The mean value of FBG (12.0 and 12.3 mmol/L) and PBG (18.4 and 17.9 mmol/L) level maybe potentially affected by two recommendations when initiating insulin. CDS and ADA recommend initiating insulin when FBG ≥ 11.1 mmol/L and random blood glucose ≥ 16.7 mmol/L for new diagnosed patients (5, 17), respectively. These two recommendations give a higher level of blood glucose for initiating insulin which may confuse physician. Our patient’s blood glucose level is just above the recommended level. On the other side, this suggests that doctors are more likely to rely on blood glucose rather than A1C level when initiating insulin therapy.

The age and history are different between patients with and without A1C indicate that older patients and that with longer history are prone to initiate insulin based on blood glucose.

There are several limitations in our study. First, patients use their own and different glucometers which could affect the results. Second, our FBG and PBG are one reading, a mean value for FBG or PPG would be better.

In summary, our results demonstrated that initiating insulin based on FBG or PBG is a common clinical practice, at least in China. Moreover, since it is easier to obtain than A1C, it can be a simple and effective way to overcome clinical inertia for initiating insulin. Maybe the combination of both A1C and FBG and PBG will give the best guidance.

## Data availability statement

The raw data supporting the conclusions of this article will be made available by the authors, without undue reservation.

## Ethics statement

The studies involving human participants were reviewed and approved by The academic ethics review boards of the Second Affiliated Hospital of Guangzhou Medical University. The patients/participants provided their written informed consent to participate in this study.

## Author contributions

WL and DY contributed to the design of the study, data analysis, and manuscript preparation and overviewing. Other co-authors participated in the patient recruitment, implementation of the study, and manuscript overviewing. All authors contributed toward data analysis, drafting and critically revising the paper and agree to be accountable for all aspects of the work. All authors contributed to the article and approved the submitted version.

## Funding

This work was supported by National Natural Science Foundation of China (grants number: 81800682 to YL), the Medical and Health Project of Guangzhou (grants number: 20201A011079 to YL), and Guangzhou Science and Technology Project (grants number: 202201020550 to YL).

## Conflict of interest

The authors declare that the research was conducted in the absence of any commercial or financial relationships that could be construed as a potential conflict of interest.

## Publisher’s note

All claims expressed in this article are solely those of the authors and do not necessarily represent those of their affiliated organizations, or those of the publisher, the editors and the reviewers. Any product that may be evaluated in this article, or claim that may be made by its manufacturer, is not guaranteed or endorsed by the publisher.
